# Predictor of cardiovascular risks in end stage renal failure patients on maintenance dialysis

**DOI:** 10.12669/pjms.316.8039

**Published:** 2015

**Authors:** Christopher Thiam Seong Lim, Xian Hui Yap, Kuet Jun Chung, Mohamad Azrul Khalid, Norhayati Yayha, Latiffah A. Latiff, Bak Leong Goh

**Affiliations:** 1 Christopher Thiam Seong Lim, Department of Medicine, Faculty of Medicine and Health Sciences, Universiti Putra Malaysia, Malaysia; 2 Xian Hui Yap, Department of Medicine, Faculty of Medicine and Health Sciences, Universiti Putra Malaysia, Malaysia; 3 Kuet Jun Chung, Department of Medicine, Faculty of Medicine and Health Sciences, Universiti Putra Malaysia, Malaysia; 4 Mohamad Azrul Khalid, Department of Medicine, Faculty of Medicine and Health Sciences, Universiti Putra Malaysia, Malaysia; 5 Norhayati Yayha, Department of Medicine, Faculty of Medicine and Health Sciences, Universiti Putra Malaysia, Malaysia; 6 Latiffah A. Latiff, Department of Medicine, Faculty of Medicine and Health Sciences, Universiti Putra Malaysia, Malaysia; 7 Bak Leong Goh, Department of Nephrology, Serdang Hospital, Malaysia

**Keywords:** CVD risk, End Stage Renal Failure (ESRF), Haemodialysis, Peritoneal dialysis

## Abstract

**Objective:**

Cardiovascular disease (CVD) is the main cause of morbidity and premature mortality in end stage renal failure patients (ESRD) receiving dialysis. The aim of our study was to evaluate the impact of various risk factors in this group of high CVD risk patients in local population.

**Methods:**

We carried out a cross-sectional retrospective study in a single hospital. A total of 136 ESRF patients, consisted of 43 haemodialysis (HD) and 93 continuous ambulatory peritoneal dialysis (CAPD) patients, were recruited and followed up for 36 months duration. Midweek clinical and laboratory data were collected. The occurrence of existing and new CVD events was recorded.

**Results:**

Multiple Logistic Regression showed pre-existing cardiovascular event (odds ratio, 4.124; 95% confidence interval [CI], 0.990 to 17.187), elevated total cholesterol level (odds ratio, 0.550; 95% CI, 0.315 to 0.963), elevated serum phosphate level (odds ratio, 5.862; 95% CI, 1.041 to 33.024) and elevated random blood glucose level (odds ratio, 1.193; 95% CI, 1.012 to 1.406) were significantly associated with occurrence of CVD events.

**Conclusions:**

History of cardiovascular event before the initiation of dialysis, elevated level of serum phosphate and random blood glucose levels are the risk factors of CVD whereas paradoxically a high total cholesterol level has CVD protective effect towards the ESRF patients.

## INTRODUCTION

CKD is an independent risk factor cardiovascular morbidity and mortality (CVD), even after adjustment for traditional and non-traditional risk factors. CVD events like cerebrovascular accident (CVA), ischeamic heart disease (IHD), congestive cardiac failure (CCF) and peripheral vascular disease (PVD) are much higher in CKD population.^1^

Traditional risk factors like hypertension, diabetes, hyperlipideamia and male gender cannot explain the abnormally high incidence of CVD in ESRF patients. The mortality rate for dialysis patients is abnormally high, up 10 to 30 times higher than the matched population.^1^

Several theories have been proposed over the last decade over the role of traditional and non-traditional contributing factors in accelerating CVD in ESRF population. However all these studies were done abroad with considerable different ethnic origin.

In this study, we have undertaken a local study to evaluate whether we could predict the CVD risk for our local ESRF patients by using the routine parameters that are easily available in our day to day clinical practice. We hope by identifying these factors, we can institute strategy that will lessen the CVD risks in ESRF patients.

## METHODS

This was a retrospective, cross-sectional, single center study. Incident ESRF patients undergoing Haemodialysis (HD) and Chronic Ambulatory Peritoneal Dialysis (CAPD) at Serdang Hospital from April 2006 until April 2009 were recruited by means of universal sampling. ESRF patients were defined as those having Glomerular Filtration Rate (GFR) < 15ml/min and have been started on dialysis for three months duration. The renal replacement therapy is of either haemodialysis or peritoneal dialysis. ESRF patients who had changed their dialysis modalities, either from HD to CAPD or vice versa or new ESRF of less than three months were excluded and those who had renal transplantation done were excluded.

Data were collected by three trained researchers that reviewed the hospital electronic medical records of the recruited ESRF patients. The collected data that were entered into a proforma which capture data like socio-demographic factors, baseline medical history, essential routine laboratory parameters and the occurrence of new CV events.

This study was non-interventional and did not influence the nature of treatment received by the subjects. Institutional review board (IRB) approval was obtained and either the patients or patients’ closest available next-of-kin were approached in all cases for informed consent using IRB-approved informed consent form. This was an investigator- initiated study.

Data was analysed using SPSS version 18.0. The normality of continuous data was tested using Kolmogorov-Smirnov test. χ² test was used to test for the association of categorical data and p value ≤0.05 was considered significant. For continuous data which was not normally distributed, Mann-Whitney U test was used to test for the association. For dichotomous outcomes such as the CV events, Multiple Logistic Regression was used to determine the absolute risk on incidence.

## RESULTS

A total of 136 of dialysis patients were recruited into the study. Table-I shows the demographic, laboratory parameters and the new CV events. Majority of the patients were within the 65-74 age group. The most common cause of ESRF in our center was diabetic nephropathy. The co-morbidities documented were hypertension (96.5%), diabetes (66.2%), hyperlipidemia (58.1%) and pre-existing cardiovascular event (31.6%).

**Table-I:** Summary demographic, laboratory parameters, and cardiovascular events (n=136).

Variables	Frequency (%)	Mean	Median ± IQR
Socio-demographic Factors			
Age		50.96	55 ± 20
15-34	22 (16.2)		
35-54	44 (32.4)		
55-74	66 (33.1)		
≥75	4 (2.9)		
Gender			
Male	68 (50.0)		
Female	68 (50.0)		
Race			
Malay	70 (51.5)		
Chinese	48 (35.3)		
Indian	18 (13.2)		
Causes of ESRD			
DM	45 (33.1)		
DM + HPT	32 (23.5)		
Unknown	24 (17.6)		
HPT	15 (11.1)		
GN	2 (1.5)		
SLE	1 (0.7)		
Co-morbidities			
HPT	130 (95.6)		
DM	90 (66.2)		
Hyperlipidaemia	79 (58.1)		
Pre-existing CV event	43 (31.6)		
Essential Laboratory Parameters				
Lipid Profile			
Total Cholesterol (mmol/L)		5.17 ± 1.70	
LDL (mmol/L)			3.11 ± 1.47
HDL (mmol/L)			1.15 ± 0.40
Triglyceride (mmol/L)			1.86 ± 1.29
Serum Calcium (mmol/L)			2.19 ± 0.31
Serum Phosphate (mmol/L)		1.42 ± 0.53	
Random Blood Glucose (mmol/L)		8.18 ± 5.50	
Haemoglobin (g/dL)			10.40 ± 2.40
Cardiovascular Event			
CV event (overall)	37 (27.2)		
CVA	12(8.8)		
IHD	7(5.1)		
CCF	2(1.5)		
PVD	16(11.8)		

*DM: Diabetes Mellitus, HPT: Hypertension, GN: Glomerulonephritis, SLE: Systemic Lupus Erythematosus, LDL: Low density lipoprotein, HDL: High density lipoprotein, CVA: Cerebral vascular accident, IHD: Ischaemic heart disease, CCF: congestive cardiac failure, PVD: peripheral vascular disease.

In the bivariate analysis results, there were significant association between hypertensive nephrosclerosis (p=0.040), diabetes mellitus (p=0.050) and pre-existing CVD (p=0.024) with the occurrence of new CVD events (Table-II). As for laboratory tests, there were significant associations between LDL (p=0.043) and random blood glucose (p=0.014) level with the occurrence of new CVD events (Table-III).

**Table-II:** Bivariate analysis of baseline medical history with cardiovascular events.

Baseline Medical History		CV Events				χ^2^ value/Fisher’s Exact Test	p value
		Yes		No			
		n	%	n	%		
*Causes of ESRD*							
Unknown	Yes	1	(4.2)	23	(95.8)	^b^	0.304
	No	15	(13.4)	97	(86.6)		
DM	Yes	12	(14.8)	69	(85.2)	1.795^a^	0.180
	No	4	(7.3)	51	(92.7)		
GN	Yes	1	(25.0)	3	(75.0)	^b^	0.397
	No	15	(11.4)	117	(88.6)		
SLE	Yes	0	(0)	1	(100.0)	^b^	1.000
	No	16	(11.9)	119	(88.1)		
Polycystic kidney disease	Yes	0	-	0	-	-	-
	No	16	(11.8)	120	(88.2)		
Obstructive nephropathy	Yes	0	(0)	5	(100.0)	^b^	1.000
	No	16	(12.2)	115	(87.8)		
Hypertension	Yes	10	(18.9)	43	(81.1)	4.221^a^	0.040*
	No	6	(7.2)	77	(92.8)		
Others	Yes	0	(0)	12	(100.0)	^b^	0.359
	No	16	(12.9)	108	(87.1)		
*Co-morbidities*							
Hypetension	Yes	15	(11.5)	115	(88.5)	^b^	0.535
	No	1	(16.7)	5	(83.3)		
Diabetes Mellitus	Yes	14	(15.6)	76	(84.4)	3.684^a^	0.050*
	No	2	(4.3)	44	(95.7)		
Hyperlipidaemia	Yes	11	(13.9)	68	(86.1)	0.847^a^	0.357
	No	5	(8.8)	52	(91.2)		
Pre-existing CV	Yes	34	(79.1)	9	(20.9)	5.089^a^	0.024*
	No	86	(92.5)	7	(7.5)		

* p≤0.05 is significant

a χ^2^ Test

b Fisher’s Exact Test.

**Table-III:** Bivariate analysis of laboratory parameters and cardiovascular events.

Lab Parameters	CV Event	Median ± IQR	Mean Rank	Z value	p value
Total Cholesterol (mmol/L)	Yes	4.45 ± 1.65	51.78	-1.807	0.071
	No	5.21 ± 1.65	70.73		
LDL (mmol/L)	Yes	2.51 ± 1.31	49.81	-0.202	0.043*
	No	3.12 ± 1.51	70.99		
HDL (mmol/L)	Yes	1.17 ± 0.37	76.16	-0.828	0.408
	No	1.12 ± 0.41	67.48		
TG (mmol/L)	Yes	1.94 ± 0.81	65.91	-0.280	0.779
	No	1.84 ± 1.31	68.85		
Serum Calcium (mmol/L)	Yes	2.19 ± 0.25	77.69	-0.993	0.321
	No	2.19 ± 0.32	67.28		
Serum Phosphate (mmol/L)	Yes	1.57 ± 0.50	82.38	-1.500	0.408
	No	1.40 ± 0.55	66.65		
Random Blood Glucose (mmol/L)	Yes	9.46 ± 5.47	91.25	-2.459	0.014*
	No	7.72 ± 5.25	65.47		
Haemoglobin (g/dL)	Yes	10.30 ± 1.73	72.91	-0.476	0.634
	No	10.40 ± 2.75	67.91		

* p≤0.05 is significant Mann-Whitney U test.

Statistical analysis via multiple logistic regression showed history of pre-existing CV event (p=0.050), serum phosphate level (p=0.045) and random blood glucose level (p=0.035) were associated with significant increased of developing new CVD (Table-IV). Interestingly a high total cholesterol level with associated with a significant fewer CV events (P=0.035). There is no correlation between CVD events and serum haemoglobin and calcium level. Other comorbidities and aetiology of ESRF do not seem to have any correlation either with CVD events.

**Table-IV:** Multivariate analysis.

Variables	B	p value	Exp (B)	95.0% C.I. for Exp (B)	
				Lower	Upper
*Socio-demoraphic Factor*					
Gender	-0.415	0.584	0.661	0.149	2.920
*Causes of ESRD*					
Diabetes mellitus	-0.893	0.378	0.409	0.056	2.981
Hypertension	1.224	0.068	3.402	0.913	12.685
*Co-morbidities*					
Hypertension	-1.487	0.300	0.226	0.014	3.769
Diabetes mellitus	1.704	0.170	5.498	0.482	62.665
Hyperlipidaemia	-0.227	0.762	0.797	0.184	3.455
Pre-existing CV event	1.417	0.050*	4.124	0.990	17.187
*Essential Lab Parameters*					
Total cholesterol	-0.597	0.036*	0.550	0.315	0.963
Serum calcium	2.128	0.159	8.397	0.434	162.481
Serum phosphate	1.769	0.045*	5.862	1.041	33.024
Random blood glucose	0.177	0.035*	1.193	1.012	1.406
Haemoglobin	0.104	0.624	1.109	0.733	1.679
*Constant*	-9.400	0.016	0.000		

* p≤0.05 is significant; Nagelkerke R Square=0.353

## DISCUSSION

There is abundance of data which demonstrated the association of ESRF and CVD.^1^ The overall prevalence of CVD in our center was 31.6% with incidence rate of 27.2%.

The aetiology of ESRF appeared to have positive correlation with CVD where diabetic nephropathy and hypertensive nephrosclerosis has the worst outcome as shown in the bivariate analysis. In a large, prospective cohort study that included 12 550 adults, the development of type II diabetes was almost 2.5 times as likely in persons with hypertension than in their normotensive counterparts. This, in conjunction with considerable evidence of the increased prevalence of hypertension in diabetic persons, suggests that these 2 common chronic diseases frequently coexist. Moreover, each pathophysiological disease entity, although independent in its own natural history, serves to exacerbate the other.^2^

The bivariate analysis also demonstrated that an elevated random blood glucose level or low density lipoprotein (LDL) level significantly increased the cardiovascular risk. However further multivariate analysis demonstrated the inverse relationship with total cholesterol level and CVD. Our study supported the hypothesis whereby a lower plasma total cholesterol level in ESRF patient was associated with a significantly higher risk of death, possibility due to confounding protein-energy wasting or ‘time discrepancy of competing risks’ in which hypercholesterolaemia is beneficial only in the short term, while it worsens survival over a long-term interval.^3^ Cholesterol level reflects the nutritional status and well being of the ESRF patient a low cholesterol level is a results of malnutrition and on-going inflammation.^4^

The multivariate analysis again reconfirmed the increase cardiovascular risks with elevated random sugar level and phosphate level. In a recently published meta-analysis and systemic analysis, a high glycated haemoglobin level significantly increased the mortality risk in ESRF patient on dialysis. Poor glycemic control might result directly in macrovascular complications, possibly secondary to the generation of advanced glycation end products (AGEs), and hence shorten survival of these patients.^5^

In ESRD patients, as glomerular filtration rate decreases, there will be accumulation of inorganic phosphate which leads to stimulation of parathyroid hormone (PTH) in an attempt to restore the serum phosphorus and calcium homestasis. We found that hyperphosphatemia significantly increased risk of cardiovascular events by 5.8 times. Inadequate control of serum phosphorus contributed to elevated insoluble calcium-phosphorus (Ca x P) product, which lead to cardiovascular calcification, and accelerated CVD manifestation.^6^ In our study, there was no association between serum calcium level and occurrence of cardiovascular event in dialysis patients. The presence and extent of vascular calcifications rather than serum calcium were strong predictors of cardiovascular mortality.^6^

In our study, there were 31.6% dialysis patients who had pre-existing cardiovascular event prior to initiation of dialysis treatment. In both bivariate and multivariate analysis, pre-existing CVD was statistically associated with cardiovascular event. Those with pre-existing cardiovascular disease also has 4.1 times risk of developing further cardiovascular complications. The pre-existence of CVD appeared to be a strong predictor for CVD event. This finding concurred with the findings done in Canada by Kalantar-Zadeh.^7^

Majority of our patient has anaemia of chronic disease. Generally, patients with ESRD will develop anaemia mainly due to the inability of the kidneys to secrete enough erythropoietin to stimulate adequate hematopoiesis.^8^ Apart from erythropoietin deficiency, uremic-induced inhibitors of erythropoiesis, shortened erythrocyte survival, and disordered iron homeostasis all contribute to the development of anaemia. Recent work has identified hepcidin excess as a main contributor to the disordered iron homeostasis and anemia of CKD by impairing dietary iron absorption and iron mobilization from body stores.^9^

From both the bivariate and multivariate analysis, our study showed there was no association between haemoglobin level and cardiovascular event. Patients with ESRD and mild-to-moderate anaemia, the normalization of heamoglobin levels to 13.0 to 15.0 g/dL did not reduce cardiovascular events.^10^

We acknowledge that we have not studied all the potential risk factors but rather of those which are routinely available and easily carried out in our day to day practice. Our study is also limited by the sample size and it is a single center study which may not be accurate representation of the regional trend. Nevertheless this study reassured us that routine measurement and history taking is invaluable in determining the cardiovascular risk factors.
